# Feasibility of dc vaccine combined with low dose endoxan and high dose il-2 treatment associated with taurolidine for advanced, multi-resistant melanoma patients.

**DOI:** 10.1186/2051-1426-3-S2-P209

**Published:** 2015-11-04

**Authors:** Gustavo A Moviglia, Ralf Kleef

**Affiliations:** 1 Maimonides University, Ciudad de Buenos Aires, Argentina; 2 Maimonides University, Vienna, Austria

## Background

2014 Dillman and coworkers reported 40% 5-year survival of advanced malignant melanoma (MM) patients treated with a combination of Tumor Vaccine in combination with High dose IL-2 versus 13% of similar patients treated only with High Dose of IL-2 (HD IL-2). In order to improve the efficacy of this approach and minimize the adverse effects of HD IL-2 therapy we have developed a protocol using a Dendritic Cell Vaccine challenged with autologous MM stem cells in combination with HD IL-2 and to Taurolidine (to diminish the vasculary leak syndrome).

## Methods

5 advanced and rapidly progressive MM patients, resistant to any other standard therapy were treated. 1/5 with large brain metastasis was withdrawn from the treatment after the first cycle of treatment for rapid progression of his disease. 4/5 patients received three cycles. In brief, previous to vaccination with a patient specific autologous dendritic cell vaccine patients underwent low dose endoxan 300m/m2, and were subsequently treated for 5 days with high-dose IL-2 in combination with Taurolidine as described by O'Brian et.al. 2006.

## Results

Major side effects were high temperature 4/4 and hypereosinophilia associated with itching (2/5). No other signs like severe edema, renal failure or any other life threatening condition was observed. 4/5 patients did not show any progression of their condition during the 2-3 months of observation.

## Conclusions

DC-vaccine associated to HD IL-2 + Taurolidine vaccine seems to be a feasible and low toxic treatment. Longer observation time as well as increment of the number of patients treated is necessary to confirm these preliminary findings.

